# Conceptual Combination in the LATL With and Without Syntactic Composition

**DOI:** 10.1162/nol_a_00048

**Published:** 2022-02-10

**Authors:** Alicia Parrish, Liina Pylkkänen

**Affiliations:** Department of Linguistics, New York University, New York, USA; Department of Psychology, New York University, New York, USA; NYUAD Institute, New York University Abu Dhabi, Abu Dhabi, UAE

**Keywords:** conceptual combination, syntax, semantics, magnetoencephalography

## Abstract

The relationship among syntactic, semantic, and conceptual processes in language comprehension is a central question to the neurobiology of language. Several studies have suggested that conceptual combination in particular can be localized to the left anterior temporal lobe (LATL), while syntactic processes are more often associated with the posterior temporal lobe or inferior frontal gyrus. However, LATL activity can also correlate with syntactic computations, particularly in narrative comprehension. Here we investigated the degree to which LATL conceptual combination is dependent on syntax, specifically asking whether rapid (∼200 ms) magnetoencephalography effects of conceptual combination in the LATL can occur in the absence of licit syntactic phrase closure and in the absence of a semantically plausible output for the composition. We find that such effects do occur: LATL effects of conceptual combination were observed even when there was no syntactic phrase closure or plausible meaning. But syntactic closure did have an additive effect such that LATL signals were the highest for expressions that composed both conceptually and syntactically. Our findings conform to an account in which LATL conceptual composition is influenced by local syntactic composition but is also able to operate without it.

## INTRODUCTION

Language comprehension and production require the combination of smaller units of words and morphemes into larger phrases. The last several decades of research on language processing has yielded many candidates for hubs of basic combinatorial processes. The combinatory role of the left anterior temporal lobe (LATL) has been characterized in a series of studies and appears to be conceptual in nature (e.g., Baron & Osherson, 2011; Baron et al., 2010; Blanco-Elorrieta & Pylkkänen, 2016; Kim & Pylkkänen, 2019; Poortman & Pylkkänen, 2016; Westerlund & Pylkkänen, 2014; Zhang & Pylkkänen, 2015, 2018a, 2018b), a finding also supported by studies of language deficits (e.g., Lukic et al., 2021; Mesulam et al., 2015, 2019; Wilson et al., 2014). At the same time though, the LATL also correlates with syntactic processing steps during narrative comprehension (Brennan et al., 2016; Humphries et al., 2005), though the extent to which these results may reflect semantic processing is unclear.

While these studies have concluded that the composition computed in the LATL is not itself syntactic, it leaves open the question of to what degree the computation interacts with or is dependent on syntactic processes associated with composition and phrase closure, and whether other well-studied factors such as semantic plausibility affect the LATL computation. This study explores both of these questions by systematically varying the type and validity of syntactic composition and semantic plausibility and investigating whether disruptions to these cause corresponding disruptions to *conceptual* combination, as reflected in LATL activity. We will use the term *LATL conceptual combination* to refer to the computations localized in the LATL that appear to contribute to conceptual combination. We do not mean to imply that there may not be other neural reflexes of conceptual combination or additional computational routines that achieve it (see, e.g., Boylan et al., 2015, 2017). We simply suggest that the LATL is *a* component of the process of combination. Similarly, we do not suggest that the LATL’s language function is only combinatory, given evidence for its role in single-word semantic memory (Binney et al., 2010; Lambon Ralph et al., 2009; Westerlund & Pylkkänen, 2014) and as a more general semantic hub (Lambon Ralph et al., 2017).

In contrast to the LATL’s role in conceptual, meaning-based composition, a broader range of hypotheses remain open for the left inferior frontal gyrus (LIFG) and left posterior temporal lobe (LPTL), often cited as loci of syntactic processing (Friederici, 2011; Grodzinsky & Santi, 2008; Hagoort, 2005, 2017; Matchin & Hickok, 2020; Pallier et al., 2011). Certainly, all three regions are routinely implicated in normal language processing (Fedorenko & Thompson-Schill, 2014) and may even be recruited in composition in the absence of licit syntax (Mollica et al., 2020). However, the relationship between these processes and conceptual composition is an open question, as it is not clear whether conceptual combination acts on syntactically licit phrases during sentence processing, or if it functions fully independently of syntactic input. Similarly, the role of the ventromedial prefrontal cortex (vmPFC), which has been associated with more semantic manipulations (Pylkkänen et al., 2011), in conceptual composition remains unclear. VmPFC activation sometimes patterns with the LATL in showing greater activation for phrases compared to single words or lists and sometimes fails to show such an effect.

Thus this study fills in a gap in the existing literature, by asking, in a highly controlled way, to what degree conceptual combination effects are modulated by syntactic and semantic effects, and to what degree the output of conceptual combination similarly affects other processes. Our main focus was to test whether combinatory effects in the LATL can be obtained independent of syntax and, conversely, whether there are other regions not sensitive to manipulations of conceptual combination that are still modulated by syntactic differences.

**Figure 1.**
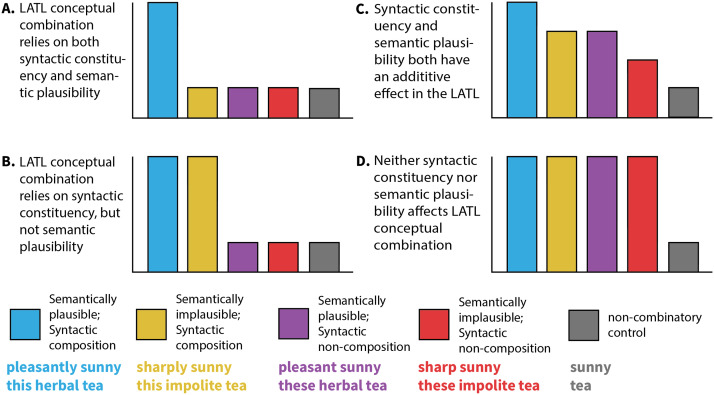
Diagram of predicted results from the four primary hypotheses. Every hypothesis predicts a difference between the non-combinatory control and the plausible composition condition.

We first address the question of whether conceptual combination relies on syntactic constituency by creating minimal pairs of phrases that have conceptual structures that are as similar as possible but vary in whether syntactic composition is possible. *Perfect* minimal pairs of this type are not possible in English, as changes to the structure very often cause a change in meaning. However, within the realm of modifiers, we have the ability to create near minimal pairs of this type, as the addition of the morpheme *-ly* can turn an adjective into an adverb, but keeps the conceptual content of the word relatively unchanged (e.g., *pleasant* and *pleasantly* have similar conceptual content). We exploit this property of modifiers to create near minimal pairs for our *adjective manipulation* that share conceptual content but differ in whether syntactic composition is possible by using an adjective as a target word, comparing activation on an adjective like *sunny* following either *pleasantly* (forming *pleasantly sunny*, where there is local syntactic composition) or following *pleasant* (forming *pleasant*, *sunny*, where this is no syntactic composition between the two modifiers). In both cases, the phrase is followed by a noun (e.g., *day*) to form a reasonable English phrase. If conceptual composition relies on syntactic composition to some degree, as would be the case if LATL composition operates on the outputs of a licit syntactic parse, then we expect to see greater activation on *sunny* in the phrase *pleasantly sunny* compared to *pleasant sunny* and no difference between *pleasant sunny* and a non-combinatory control (Figure 1A or 1B). However, if the process of conceptual combination is separate from or does not rely on syntactic parsing steps, then we expect both *pleasantly sunny* and *pleasant sunny* to show similarly greater LATL activation compared to a non-combinatory control (Figure 1D).

As the majority of past studies looking at LATL conceptual combination has focused on adjectival modification of nouns (e.g., Bemis & Pylkkänen, 2011; Blanco-Elorrieta et al., 2018; Poortman & Pylkkänen, 2016; Zhang & Pylkkänen, 2018a), we also introduced a *noun manipulation* into the experiment. Rather than using morphological cues to manipulate whether direct syntactic composition occurs, we created stimuli where syntactic composition between two words is not *impossible*, but rather closure of the phrase as a whole is blocked due to temporary ungrammaticality. We accomplished this by inserting a modifier between a determiner and the noun, comparing the composition effect on *tea* between *this herbal tea* (where full phrase closure is possible) to *these herbal tea* (where full phrase closure would lead to an ungrammatical parse), exploiting the property of English demonstratives that they must agree in number with the head noun. Each phrase was followed by a deverbal noun with number agreement to the determiner that caused the entire phrase to be syntactically licit (in this example, *drinker*(*s*) followed *tea*). Like with the adjective manipulation, if there is some modulation in LATL conceptual combination that comes from having a complete syntactic phrase (rather than just local syntactic composition), then we would expect to see greater activation in this region for cases like *this herbal tea* compared to *these herbal tea*. If the stronger possibility is true, that LATL conceptual combination only operates on the output of a syntactic composition mechanism, then we would further expect that *this herbal tea* would show greater activation than *these herbal tea*, relative to a non-combinatory control (Figure 1A and 1B).

In order to address the relationship between semantic plausibility and conceptual combination, we also varied whether the first modifier is likely to co-occur with or modify the target word. For the adjective manipulation, where *sunny* was the target word, we used the modifier *sharp*/*sharply*, which tends to only modify concrete rather than abstract nouns, to create the semantic implausibility (the exact cause of the implausibility varies, but was verified by a norming study described below). In the noun manipulation, we relied on animacy violations as the source of semantic implausibility. Using the above example, we compared activation on *tea* with a semantically plausible modifier (*herbal*) to activation on *tea* with an implausible modifier (*impolite*). In the implausible condition, it was always the case that the modifier could licitly compose with the following deverbal noun (*drinker*), leading to a globally plausible sentence.

If LATL conceptual combination relies on a meaningfully licit parse being available for the phrase, then we would expect to see greater activation in regions previously identified as participating in combinatory processes for the plausible condition, with the implausible condition patterning with the non-combinatory control (compatible with Figure 1A). Note, however, that a “meaningfully licit parse” requires composition to evaluate it (though we remain agnostic about whether it requires *conceptual combination* or implicates the LATL). Indeed, under the interpretation that one can only know whether a multiword phrase is meaningful after having actually combined the words in order to assess the meaning (Pylkkänen et al., 2009), then any effect of the plausibility manipulation on conceptual combination would be quite unexpected. However, if conceptual combination relies on the availability of semantically compatible features, then we expect that *pleasant*(*ly*) and *herbal*, which have more compatible features with *sunny* and *tea* respectively compared to the implausible *sharp*(*ly*) and *impolite*, will elicit greater activation in the LATL. However, it is also possible that the implausibility leads to a richer semantic representation of the resulting phrase, as the concept for something that is both *sharp* and *sunny* or *a tea* that is *impolite* requires a large number of otherwise unrelated (and likely nonoverlapping) semantic features. If this is the case, then it is possible that the implausible condition will show greater activation than the plausible condition in the LATL. It is also possible that both semantic plausibility and syntactic constituency can drive LATL activation while not directly modulating the effect of conceptual combination. If these two factors can independently drive LATL activation, then we would expect to see an additive effect on activation patterns, with the highest activation for the most syntactically and semantically well-formed condition, and the lowest activation still for the non-combinatory control, with the three other test conditions falling somewhere in the middle (Figure 1C). The noun manipulation additionally controlled for potential lexical probability-related effects in this design. We created two sets of stimuli that differ based on the transitional probability (TP) of the adjective and noun. This accomplished two things: First, it ensured that we can assess whether there is an effect of the syntactic manipulation independent of the likelihood of the local combination, and second, it created a condition in which participants were biased toward preferentially interpreting the adjective noun combination as the object of the deverbal noun that follows (as in *herbal tea drinker*, which is interpreted as “one who drinks [herbal tea]”).

In creating a control condition to compare to the test conditions, we needed to ensure that the target word’s context was truly non-combinatory. This is a difficult condition to create, as conceptual combination is a basic component of language processing. As such, ensuring that the context of the critical word in the control condition is non-combinatory necessitates presenting the target word in a context where it does not have any lexical material with which to compose. Though several minimal composition studies have used a string of consonants (e.g., “xgmp”) presented prior to the target word to ensure that there is no lexical content with which to compose (e.g., Bemis & Pylkkänen, 2013), we found the paradigm with consonant strings to be highly unnatural when used in full sentences. Similarly, a random or unrelated word presented in this context would be both an unnatural and a potentially elicit attempt at composition as the participant tries to make sense of the entire sentence. Therefore, we decided to use the equivalent of single-word controls following other studies of simple two-word composition (Bemis & Pylkkänen, 2011, et seq.). In our non-combinatory control condition, the control sentences begin with the unmodified target word, *sunny* or *tea*. This condition allows us to assess whether each of the test conditions independently shows an effect of conceptual combination, as all of the test conditions have lexical material prior to the target word that could potentially trigger conceptual combination, and the primary question is whether each of these test conditions shows an increase in activation *relative to* the non-combinatory control. We find it unlikely that the presence or absence of lexical material in the pre-target word region represents a confound with respect to interpreting the activation patterns in the LATL as effects related to conceptual combination. Bemis and Pylkkänen (2011), for example, included both single-word controls (like in our study) and an additional control experiment that compared combinatory phrases in a composition task to two-word sequences in a list condition. Regardless of whether the control for the combinatory condition was a single-word control or a two-word list, the combinatory condition still showed greater activation in the LATL around 200 ms after target-word onset, indicating that prior lexical material alone is not sufficient to drive LATL conceptual combination.

In the noun manipulation, we additionally included numeral controls (*one tea*, *two tea*) based on findings in past production studies where numeral modification did not lead to an increase in LATL activity compared to a non-combinatory control (Blanco-Elorrieta & Pylkkänen, 2016; Del Prato & Pylkkänen, 2014). The purpose of this additional control was to account for the effects of a syntactic mismatch alone, without any possibility of conceptual combination related effects. Each set of stimuli in the noun condition was presented with these two additional control conditions. However, this study is the first time that a numeral condition is being tested in comprehension rather than production. In our study, the numeral controls *did* show effects consistent with conceptual combination compared to the non-combinatory control described above, and thus it was not a suitable control for analysis, so we did not include these comparisons in the main results, though we return to this issue in the discussion section.

## MATERIALS AND METHODS

### Participants

Twenty-four right-handed native speakers of North American English participated in this study. All participants were neurotypical, had normal or corrected-to-normal vision, normal hearing, reported being native speakers of English, and were naive to the purpose of the study. An accuracy threshold of 70% on the globally plausible items in the behavioral task was set to ensure that all participants were paying attention. Based on this threshold, no subjects were excluded from analysis (the lowest participant scored 74.85% accuracy, but all others were well above 80%, and mean accuracy was above 90%). One subject was excluded for average dynamic statistical parametric mapping (Dale et al., 2000) activation far exceeding the average (i.e., by more than a factor of three). All analyses were done on the remaining 23 participants.

### Materials

In order to test the independent effects of syntactic composition on processes of conceptual combination, we designed stimuli where syntactic composition is blocked, but conceptual combination is still a possibility. In the adjective manipulation, we tested this with a condition that contrasts adverb-adjective sequences (e.g., *pleasantly sunny* (*day*), where there is local combination between *pleasantly* and *sunny*) with adjective-adjective sequences (e.g., *pleasant sunny* (*day*), where there is no local combination between the two adjectives; they both modify the later *day*). In the noun manipulation, we tested this by blocking composition (i.e., merge) of a higher phrasal node through a lack of number agreement between a determiner and noun. Given a potentially combinatory phrase *herbal tea*, preceding that phrase with a number-mismatching determiner will block licit closure of the higher phrasal node (*these herbal tea, compared to the licit closure with *this herbal tea*).

It was always the case that the sentences were grammatically correct following presentation of the post-target word. For the noun + syntactic non-composition items, this meant that the agreement with the plural demonstrative (*these* or *those*) occurred on the word immediately following the target word. For the sake of consistency and to allow us to analyze effects on the post-target word, it was always the case that there was a deverbal noun following the noun target words, and we ensured that participants always preferentially interpreted the noun target word as an argument, i.e., a *tea drinker* is a person who drinks tea. Note that the syntactic composition examples are essentially garden path phrases, and that the parser must reanalyze the assumed structure in these cases once the next word is encountered. It was always the case that the sentences were grammatically correct following presentation of the post-target word, but for the syntactic non-composition condition, the phrase could not be grammatically completed at the target word.

**Figure 2.**
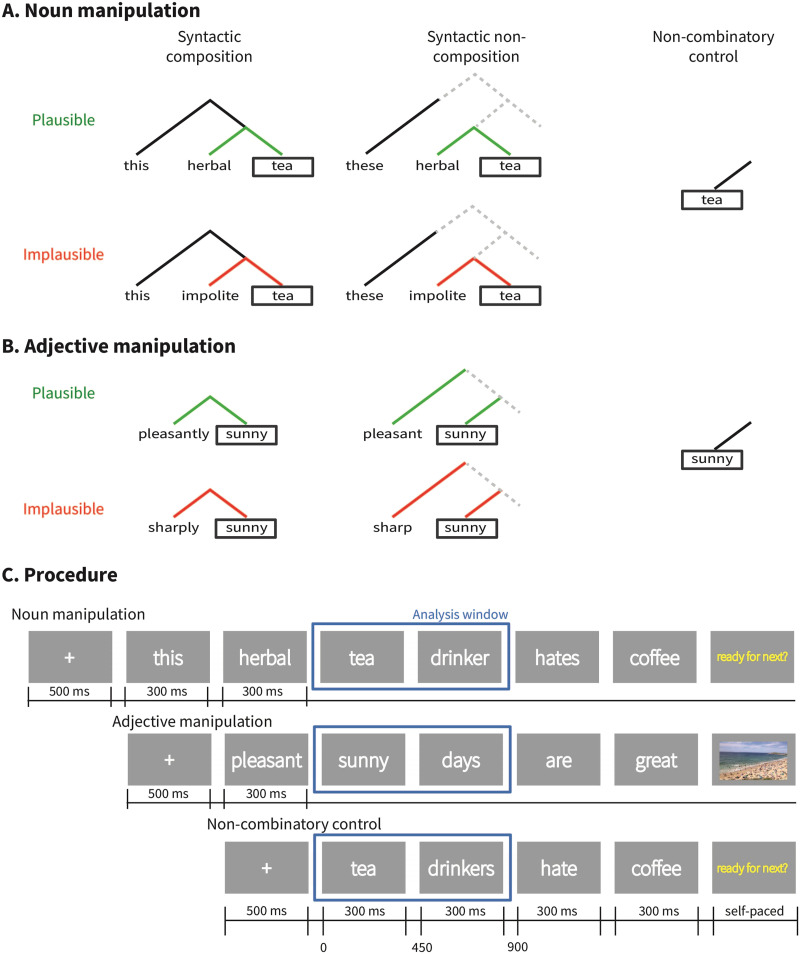
(A) and (B) show example stimuli and schematic syntactic structures used in the noun (A) and adjective (B) manipulations. Target words are boxed in each. Green lines indicate that the composition between the words is semantically plausible (e.g., *pleasantly sunny*), and red lines indicate that it is implausible given world knowledge (e.g., *sharply sunny*). Dotted lines indicate that the parser could project upcoming structure from the grammatical cues available. In the non-combinatory control condition, the target word is the initial word in the structure, so there is nothing for it to syntactically compose (or not compose) with, and there is nothing with which it can form a plausible or implausible meaning. (C) shows the timing and structure of trials in the two-word category manipulations and the control condition. Trials are arranged such that the 0 time point is the onset of the target word. The adjective manipulation shows an example of the picture verification task, and the other two examples show trials in which there was no task. In trials with a task, participants saw the “ready for next” screen after their response to the picture.

Figure 2A shows the assumed syntactic structures of each of these conditions. We additionally manipulated the semantic plausibility of each combination, yielding the full 2 × 2 × 2 paradigm of word category (adjective, noun) × semantic plausibility (plausible, implausible) × syntactic composition (composition, non-composition). The implausible condition additionally represents a meaning that is incomplete or needs to be reanalyzed after the target word, at which point phrases in the noun manipulation were globally plausible.

### Stimuli Norming

Prior to the magnetoencephalography (MEG) study, we verified (i) that the experimental stimuli represented a real contrast in plausibility between the plausible and implausible conditions, and (ii) that the syntactic manipulation in the adjective stimuli was not confounded by a difference in acceptability. We manually constructed 50 potential stimulus sets as three-word phrases for the noun manipulation with high transitional probability (e.g., *herbal*/*impolite tea drinker*), the noun manipulation with low transitional probability (e.g., *raw*/*engaged produce buyer*), and the adjective manipulation (*pleasant*(*ly*)/*sharp*(*ly*) *sunny day*). We then created the following four phrase types from each stimulus set to norm separately: (i) the full three-word phrase (e.g., *herbal tea drinker*), (ii) just the pre-target word and target word (e.g., *herbal tea*), (iii) just the target word and post-target word (e.g., *tea drinker*), and (iv) just the pre-target word and post target word (e.g., *herbal drinker*). We intentionally excluded some phrases from within the adjective manipulation that would be uninformative because the resulting phrase was grammatically illicit (e.g., *pleasantly day*) or failed to result in a complete phrase (e.g., *pleasant sunny*). This resulted in a total of 1,150 unique phrases to rate (50 noun low-TP × 7 phrase types + 50 noun high-TP × 7 phrase types + 50 adjective × 9 phrase types).

Via Amazon Mechanical Turk (MTurk; https://www.mturk.com), we conducted an acceptability judgment task in which participants rated the two- or three-word phrases on a 7-point scale, with 1 defined as “very unnatural” and 7 defined as “very natural.” Each MTurk task contained 30 to 41 items to rate, of which 5 or 8 were attention-check items with acceptability that was either very high (e.g., *large dog*) or very low (e.g., *cement laughter*). All items were presented on the same page, and all items within a task were either three-word phrases or two-word phrases. No two items derived from the same stimulus set were displayed within the same task (i.e., *herbal tea* and *impolite tea* could never appear on the same page together). Item presentation order was randomized using a Latin square design; the randomization and HTML page formatting were done using TurkTools (Erlewine & Kotek, 2016). We provide screenshots from the task instructions and the rating task in Appendix D. (Appendices for this article are located in the online Supporting Information at https://doi.org/10.1162/nol_a_00048.)

A total of 990 unique MTurk workers completed at least one online task. We used the attention-check items to identify and exclude participants who either did not understand the task or were not completing it felicitously. Accuracy criteria on the attention-check items were set as a rating of 1 or 2 on the “unacceptable” items (e.g., *cement laughter*) and a rating of 6 or 7 on the “acceptable” items (e.g., *large dog*). We applied an accuracy cutoff for each worker of 75%, which led to us excluding 85 workers (8.59%) for low accuracy. We additionally excluded 30 workers (3.03%) who indicated that they were not native speakers of English, leaving a total of 875 workers whose data were used in the study. Each item was ultimately rated by 13–18 unique MTurk workers. We also assessed inter-rater reliability with Krippendorff’s alpha (Krippendorff, 2011) to assess the degree to which raters were using the Likert scale in a similar way and found an overall agreement of α = 0.733. Within the plausible and implausible items, inter-rater reliability was at α = 0.723 and α = 0.718, respectively, indicating a reasonable degree of agreement among raters.

Of the 150 initial stimulus sets, we identified the best 35 sets within each category (noun high TP, noun low TP, and adjective). In determining which sets to include, we applied the following criteria: (i) in the noun condition, 3-word phrases had a mean rating greater than 4.5, (ii) in the adjective condition, 3-word phrases had a mean rating greater than 4 for the plausible items and lower than 4 for the implausible items, (iii) in both the noun and adjective conditions, items of the pre-target word plus the target word had mean ratings greater than 4.5 in the plausible condition and less than 3.5 in the implausible condition. This selection criteria left us with 112 viable sets (37 in noun high TP, 40 in noun low TP, and 35 in adjective), which was further reduced to the desired set size by excluding sets in which the difference between plausible and implausible items within a set was the smallest. We present the results of the norming study on the final 105 total stimulus sets that we used in the MEG study in Table 1. Most crucially, the results in Table 1 demonstrate that all stimuli used in the experiment showed a contrast in acceptability between the two-word phrases that we had labelled as plausible (e.g., *pleasantly sunny*, *herbal tea*) and those that we had labelled implausible (e.g., *sharply sunny*, *impolite tea*).

**Table 1.**
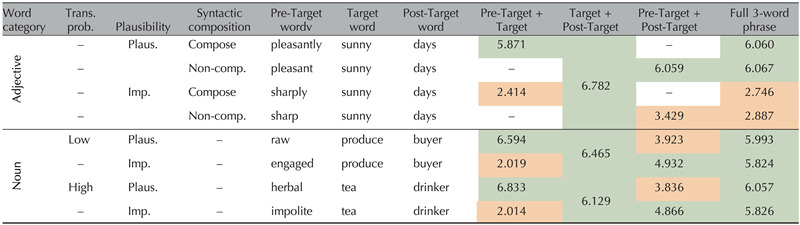
Experiment 1 results of the MTurk norming study

*Note*. Numbers represent the average raw responses from participants across all items within a given condition, as judged on a 7-point scale. The color indicates the expected acceptability rating based on the item’s label, with red being stimuli that were expected to be implausible and thus receive a low acceptability rating, and green being stimuli that were expected to be plausible and thus receive a high acceptability rating. The words under Pre-Target Word, Target Word, and Post-Target Word are examples to illustrate each experimental condition. Trans. prob = Transitional probability; Syntactic comp. = syntactic composition; Plaus. = Plausible; Imp. = Implausible; Non-comp. = Non-composition.

### Experiment Design

The full experimental design is a 2 × 2 × 2 design of word category (noun, adjective) × syntactic composition (composition, non-composition) × plausibility (plausible, implausible), with the noun manipulation additionally including a transitional probability factor (high, low). This design included one type of control item (non-combinatory) in the adjective manipulation and three types of control items (non-combinatory, numeral & syntactic composition, numeral & syntactic non-composition) in the noun manipulation. As the numeral condition turned out to not be a suitable control, we exclude this manipulation from the main results. The experiment included three groupings of 35 stimulus sets, with each set created around a single target word, and with no lexical items serving as the target word in more than one stimulus set. In the two noun manipulations (high TP, low TP), each of these sets contained seven sentences: the four test conditions, plus the three control conditions. In the one adjective manipulation, each set contained five sentences: the four test conditions, plus the one control condition. The entire experiment thus included a total of 665 sentences presented to each participant. A full list of test stimuli, along with the carrier sentences they appeared in, can be found in Appendices A and B.

These stimuli were split into 12 blocks of 54–56 trials for the experiment, with each trial consisting of a single sentence presented via rapid serial visual presentation. In assigning trials to blocks, trials were pseudo-randomized so that no two sentences (experimental or control) from the same stimulus set appeared in the same block. That is, none of the five possible sentences that used the target word *sunny* ever appeared twice in the same block. All blocks contained at least two sentences from each of the eight main test conditions (word category × semantic plausibility × syntactic composition) plus at least two controls from each word category manipulation. Trial order within blocks was fully randomized for each participant, and the order in which the blocks were presented was also fully randomized for each participant.

### Procedure

Following the guidelines set out by the Institutional Review Board of New York University, all participants gave informed, written consent before beginning the study. This experiment measured MEG responses during sentence reading and used a picture verification task. Stimuli were presented visually, one word at a time, via PsychoPy (Peirce, 2007). Each trial began with a fixation cross at the center of the screen. This cross lasted for 500 ms and was followed by a 150 ms blank screen before the first word of the stimulus sentence appeared. Words were presented with a 450 ms stimulus onset asynchrony (150 ms inter-stimulus interval). Sentences were 5–9 words in length. For 28% of the trials, the trial included an image presented after the sentence, and participants indicated with their left hand if it was a “match” or “not a match” with the sentence they just read. Each experimental block contained 14–16 task trials (out of 54–56 trials in the block), with approximately equal numbers of match and not-a-match task trials within the block. None of the images included a recognizable person (e.g., a celebrity), and none of the images contained words. As a general heuristic, participants were told to imagine whether the sentence would be a possible caption in a newspaper or online article for the picture in making their judgment. The full trial structure used for this experiment is shown in Figure 2C with an example of a trial that had a task following it. For trials where there was no task, the final word in the sentence was immediately followed by a prompt asking the participant if they were ready for the next word. The experiment lasted approximately 1 hr for each participant to complete, including 11 breaks between blocks that were each spaced approximately 5 min apart. The duration of the breaks was left up to the participants to decide.

### Data Acquisition and Processing

Participants’ headshapes were digitized using Polhemus FastSCAN system (Polhemus Inc., Colchester, USA). This headshape data, along with fiducial landmarks, was co-registered to the average brain available in FreeSurfer (Dale et al., 1999). Continuous MEG data were recorded using a 208-channel axial gradiometer whole-head system using a 1000 Hz sampling rate. All preprocessing was done using eelbrain version 0.27 (Brodbeck, 2018) and MNE version 0.16 (Gramfort et al., 2014). The MEG data was recorded with a 200 Hz low pass filter, and then filtered offline with a 0–40 Hz bandpass filter. Artifact rejection was completed with independent component analysis (ICA) for each participant to remove artifacts due to environmental noise, eyeblinks, and heartbeat. Epochs were created starting from 100 ms before the target word and extending to 1,000 ms after the target word in order to include both the target and post-target word in each epoch. Epoch rejection was done via an absolute threshold to eliminate epochs containing amplitudes exceeding 3,000 fT, as these were assumed to contain artifacts not removed with ICA. In total, 6.75% of all epochs were rejected, with no individual participant having greater than 30% of their trials rejected.

As the target word occurred in the middle of the sentence and in a different sentence position across some conditions, we de-meaned the entire epoch from 100 ms prior to the onset of the target word to 100 ms after the offset of the post-target word (for a total epoch size of 1,100 ms) rather than apply baseline correction before the target word. This allowed us to directly compare the experimental and control trials in our analysis. Channel noise covariance was estimated from 3 min of empty-room recording taken immediately prior to each participant’s recording session.

### Region of Interest Selection

We conducted analyses in four regions of interest (ROIs) selected based on prior studies showing these regions’ sensitivity to syntactic, semantic, and combinatorial manipulations. These ROIs were the anterior temporal lobe (ATL), posterior temporal lobe (PTL), inferior frontal gyrus (IFG), and vmPFC, and we analyzed both the left and right hemisphere homologues for each. For the ATL, PTL, and IFG, we based the localization of these regions on Pallier et al.’s (2011) study, taking the MNI coordinates that they identified and labelling the 30 mm regions around those points (for the ATL, we used just the temporal pole coordinates). For the vmPFC ROI, we combined the medial and lateral orbitofrontal labels from the *aparc* parcellation (Desikan et al., 2006). These ROIs and their exact coordinates are summarized in the table in Appendix C.

### MEG Analysis

All planned analyses were conducted using temporal cluster permutation tests (Maris & Oostenveld, 2007) within each predetermined ROI and time window or spatiotemporal cluster permutation tests across the whole brain. For assessing combinatory effects, we conducted a 2 × 5 ANOVA of word category × condition, and we analyzed the window that spanned from 100–300 ms after presentation of the target word and post-target word, following the findings of previous studies that identified the peak of combinatorial activity to begin around 200 ms post-stimulus onset in visually presented comprehension tasks (Bemis & Pylkkänen, 2012). We expanded this window to 100–450 ms for 2 × 2 × 2 comparisons of word category × semantic plausibility × syntactic composition (no control items) to take into account additional syntactic or plausibility-related effects that extend beyond 300 ms.

For the temporal cluster permutation test, we averaged over the sources in each ROI at each time point, and those time courses were used as input to the cluster-based permutation test. We computed an *F* statistic for the ANOVAs, and used cluster thresholds set to a minimum temporal window of at least 25 ms and formed from statistics corresponding to a *p* value of less than 0.05. Each test used 10,000 permutations. To correct for multiple comparisons across eight different ROIs, we assessed significance with the Bonferroni corrected *p* value (Rice, 1989) to take into account multiple tests against the same null hypothesis (Cabin & Mitchell, 2000).

As our ROIs were selected based on composition studies, and our stimuli included both plausibility and syntactic manipulations that may not be fully captured in these ROIs, we conducted exploratory spatiotemporal cluster permutation tests for the 2 × 2 × 2 comparison of word category × semantic plausibility × syntactic composition and for the 2 × 2 comparison of transitional probability × syntactic composition. In each case, we computed an *F* statistic for these comparisons, with minimum cluster thresholds of 25 ms in duration with at least 10 contiguous sources corresponding to an uncorrected *p* value of less than 0.05, with 10,000 permutations.

## RESULTS

### Effects of Conceptual Combination as Reflected by Comparison to Non-Combinatory Controls

**Figure 3.**
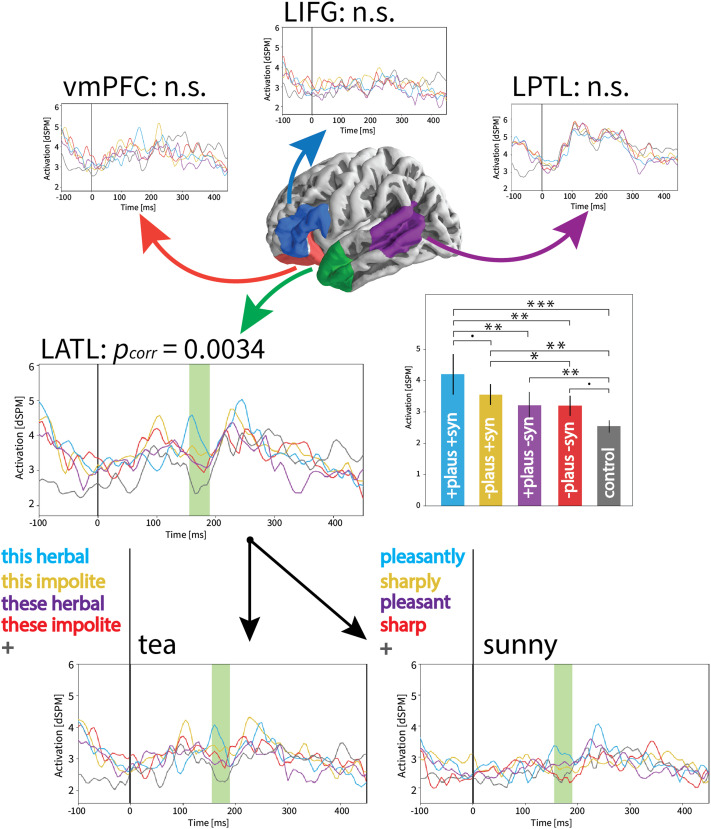
Main effect of condition in all LH ROIs from the 2 × 5 ANOVA crossing word category and condition. Only the LATL showed a reliable effect, with each of the four test conditions showing greater amplitudes than the control for both adjectives and nouns (i.e., no interaction with word category). Thus the possibility of conceptual combination appears to increase LATL activity even in the absence of local syntactic composition or plausibility. The corresponding RH analysis yielded no significant effects. ***p < 0.001, **p < 0.01, *p < 0.05, ●*p* < 0.1.

On the target word (*sunny*, *tea*), we observed a main effect of condition (*p*_*corr*_ = 0.0034) in the LATL characterized by increased activation for all four test conditions compared to the non-combinatory control, and the highest activation for the plausible + syntactic composition condition. The identified cluster spanned from 155–190 ms post-stimulus onset. None of the other seven ROIs showed a significant main effect of condition. All four test conditions appeared to have greater average activation in the identified time window compared to the control. Follow-up pairwise *t* tests showed that this difference was significant in three of the four test conditions (all *p*s < 0.005), with only the implausible + syntactic non-composition condition not passing the significance threshold (*p* = 0.064). As these were follow-up tests from a significant main effect and were conducted within a single identified ROI, they were not further controlled for multiple comparisons. There were additional significant differences between the plausible + syntactic composition condition and the two syntactic non-composition conditions (*p*s < 0.01). These results are summarized in Figure 3, where we also show that this effect trends in the same direction for both the noun manipulation and the adjective manipulation (noun and adjective plots are for visualization purposes, as there was no interaction of word category by condition, so we did not run comparisons within each word category manipulation).

Additionally, we tested the *one tea* and *two tea* numeral controls for the noun manipulation, but we did not observe any differences between these control sentences and the test sentences in any of our ROIs. Nor did we observe any patterns of marginally significant differences, even when applying a looser cluster threshold of *p* < 0.1.

### Factorial Analysis With Plausibility and Syntactic Composition

We compared the test conditions in a 2 × 2 × 2 ANOVA of word category × syntactic composition × plausibility. We observed a significant main effect of syntactic composition in the LATL, characterized by an increase for the syntactic composition condition compared to syntactic non-composition (*p*_*corr*_ = 0.0496). The temporal cluster identified extended from 150–190 ms after onset of the target word. Left vmPFC showed a non-significant trend toward the same effect of greater activation in the syntactic composition condition compared to the syntactic non-composition condition, with a cluster that extended 150–190 ms after target word onset (*p*_*corr*_ = 0.0768). Though the effect in LIFG was in the same direction as the LATL effect, the strongest cluster was not significant (160–190 ms, *p*_*corr*_ = 0.2568). After multiple comparison correction, there were no significant main effects of syntactic composition on the post-target word. For both the target word and post-target word, no significant interactions between syntactic composition and either word category or semantic plausibility were identified in any of the ROIs, and no significant effects of syntactic composition were observed in the right hemisphere ROIs.

We observed a significant main effect of semantic plausibility in the LPTL, with an identified temporal cluster that extended 320–370 ms after onset of the target word (*p*_*corr*_ = 0.012). Similar trends were observed in LIFG (*p*_*corr*_ = 0.02, 325–370 ms) and LATL (*p*_*corr*_ = 0.0856, 330–360 ms). There were no main effects of semantic plausibility on the post-target word. For both the target word and post-target word, no significant interactions between semantic plausibility and either word category or syntactic closure were identified in any of the ROIs, and no significant effects of semantic plausibility were observed in the right hemisphere ROIs. We also observed a main effect of word category that persisted even after controlling for the greater number of noun trials compared to adjective trials on the target word in the left vmPFC (170–195 ms after presentation of the target word, *p*_*corr*_ = 0.028), with greater activation for the adjectives (e.g., *sunny*) compared to the nouns (e.g., *tea*). There was also a significant difference on the post-target word that was characterized by greater amplitude in the noun manipulation (e.g., *drinker*(*s*)) compared to the adjective manipulation (e.g., *days*). This effect was significant in the LPTL (235–280 ms after presentation of the post-target word, *p*_*corr*_ = 0.0064). No right hemisphere ROIs showed a significant main effect of word category on either the target word or post-target word, and there were no ROIs that showed a significant interaction of word category by condition.

### Exploratory Spatiotemporal Analyses

**Figure 4.**
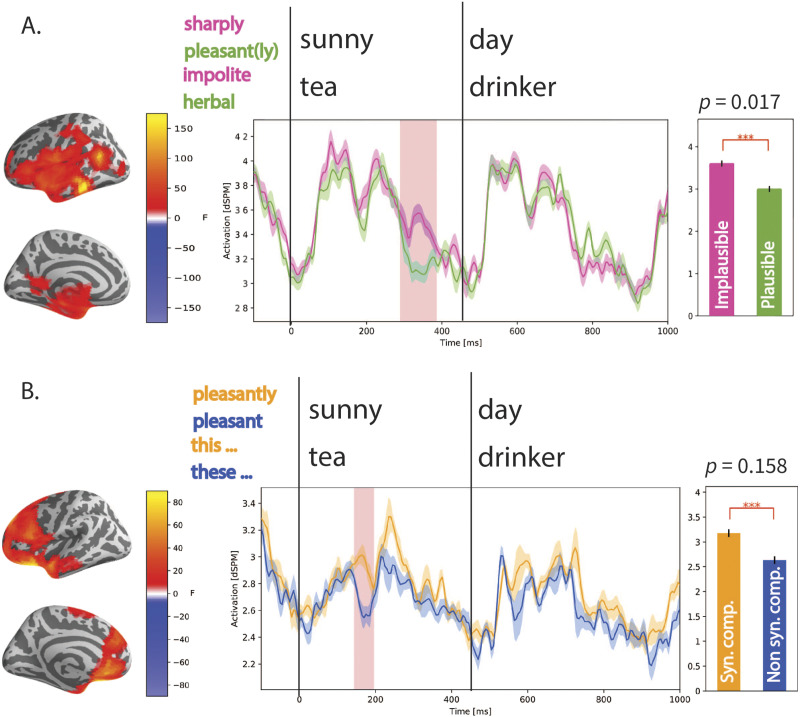
Main effects of semantic plausibility (A) and syntactic composition (B). Implausible nouns and adjectives showed increased amplitudes at 300–390 ms after stimulus onset, widely distributed across the left hemisphere, but most strongly localizing in the middle temporal lobe, consistent with previous localization of N400-type plausibility effects (Lau et al., 2008; Maess et al., 2006). The main effect of syntactic composition shown in (B) was only a trend (*p* = 0.158), but showed greater activation for the conditions where the syntax fully composes. The identified cluster ranges from 150 to 200 ms after stimulus presentation and localizes most strongly near the vmPFC, but has a fairly broad frontal distribution. There were no significant interactions between any of the factors (word category × semantic plausibility × syntactic composition) in either the target word window (*sunny*/*tea*) or the post-target word window (*day*/*drinker*). Significant main effects of word category (not shown) were all restricted to the left hemisphere after controlling for differences in the number of trials.

As the selected ROIs were not particularly well-suited to identify plausibility effects, and because the pattern of significant and trending results for both the plausibility manipulation and the syntactic manipulation indicated that the associated activation patterns may be more distributed, we conducted an exploratory spatiotemporal test on the whole brain using the full 2 × 2 × 2 design. In Figure 4, we show the most significant spatiotemporal clusters identified for these factors. Figure 4A shows the significant cluster associated with the semantic plausibility manipulation, where the implausible condition shows greater activation than the plausible condition. The cluster is significant in a time window from 300–390 ms after target word onset (*p* = 0.017), and the cluster localizes most strongly to the left middle temporal lobe, though the difference in activation is fairly distributed. Figure 4B shows a non-significant spatiotemporal cluster that was identified as the strongest effect of the syntactic manipulation (*p* = 0.158). As these analyses were conducted over the whole brain rather than within pre-selected ROIs, they were corrected within the clustering threshold and not via the post-hoc Bonferroni correction used for the ROI analyses.

No significant clusters were observed in right hemisphere regions, and no significant interactions were observed between any of the factors. On the post-target word (*drinker*(*s*), *day*), we did not observe any significant effects for semantic plausibility or syntactic closure. There were two significant clusters associated with word category. Both clusters extended for the full analysis window of 100–400 ms after the onset of the post-target word, with one cluster encompassing the entire left hemisphere and the other cluster encompassing most of the right hemisphere, making interpretation of this significant result uninformative.

### Effects of Transition Probability in the Noun Manipulation

We conducted two spatiotemporal cluster permutation tests with 2 × 2 factorial analyses of transition probability × condition and transition probability × syntactic composition to assess if conceptual combination was modulated by the local probability of the target word. We did not find any significant main effects of transition probability or any interactions on the target word or the post-target word. The only cluster identified was a non-significant effect on the post-target word (*p* = 0.191) that was broadly distributed across frontal and temporal regions of the left hemisphere, but localized most strongly around the STG, showing greater activation for the high transition probability condition (*herbal tea drinker*) compared to low (*raw produce buyer*). No significant clusters were found in the right hemisphere.

## DISCUSSION

We observed that the LATL conceptual composition effect occurred both in the absence of syntactic composition and in the absence of a plausible semantic interpretation. This finding suggests, first, that LATL conceptual combination is a processing step that is not dependent on the syntactic parse. However, we cannot conclude that syntactic information is irrelevant for the LATL or for conceptual combination more generally, as we observed an effect of the syntactic manipulation in the LATL in a similar time window as the conceptual combination effect, such that syntactic composition on a given word corresponded to greater activation. In other words, we effectively found a three-way distinction, such that there is higher activation on the target word *sunny* in phrases like *pleasantly sunny* compared to *pleasant sunny*, and both of these phrases show greater activation than the non-combinatory control *sunny*. While in the adjective manipulation we could directly manipulate whether local syntactic merge occurred, the difference was subtler in the noun manipulation. The three-way contrast in that case would be that *this herbal tea* shows greater activation on *tea* compared to *these herbal tea* (both of which show greater activation than the non-combinatory control *tea*), even though in both cases there is a local combination that takes place, namely between *herbal* and *tea*. As there was no interaction in the conceptual combination effects with word category, and as the same pattern of activation was present within the noun and adjective manipulations, we conclude that full licit phrase closure for the DP (determiner phrase), which is possible in *this herbal tea* but not *these herbal tea* is also a driver of whatever syntactic parsing step is responsible for the observed increases in LATL activation.

There are at least two plausible mechanistic accounts of this syntactic effect co-occurring with LATL conceptual combination: (i) an earlier processing step that involves syntactic composition of constituents feeds into the LATL during a time window consistent with conceptual combination, or (ii) syntactic feature information is composed in tandem with conceptual composition, and the highest increase in the syntactic composition condition is an additive effect of two separate processes. These two possibilities cannot be distinguished on the basis of just this study, but one aspect of the design hints toward the second possibility. Our second control condition in noun manipulation used numeral modification as an additional less conceptual but combinatory condition based on the findings of a production study. However, as we did not observe any differences between the numeral controls and the test conditions, we consider it possible that, at least for comprehension, numerals may to some extent engage the LATL conceptual combinatory hub. The syntactic number features *do* need to be checked, and if this process implicates the LATL, then it may be that the presence of this effect is the reason for the lack of a finding when comparing *one tea* to *this herbal tea*. It is also possible that in comprehension, numerals *are* treated as conceptually composing with the nouns that they modify, whereas production does not engage such a process. Further study is needed to disentangle these options.

Based on previous studies that have found that syntactic composition drives activity in the left PTL rather than the left ATL (Flick & Pylkkänen, 2020; Matchin & Hickok, 2020), it is perhaps surprising that our syntactic manipulations did not show any significant effects in the left PTL. However, the syntactic manipulation in this study was much subtler than that used by Flick & Pylkkänen, who were contrasting predication with modification. All of our cases are strictly modificational, and thus might not be enough of a syntactic distinction to drive measurable LPTL activity. It is worth noting, however, that the time course at which we measured syntactic modulations in the LATL is roughly aligned with the time course with which Flick & Pylkkänen observed an effect of their syntactic manipulation in the LPTL, possibly pointing toward these reflecting similar underlying computations.

Similarly, the lack of a significant effect of the syntactic manipulation in the LIFG may be surprising given the long history of this region’s association with syntactic processes (e.g., Friederici et al., 2006). Again, a number of studies that find support for a syntactic role for the LIFG are comparing much more distinct computations than what this study used, such as word lists vs. full sentences (Friederici et al., 2000, i.a.; Zaccarella et al., 2017). In a more directly comparable study, Schell et al. (2017) found an increase in LIFG activation for minimal phrases like *this ship* compared to single word controls using fMRI. The difference between their findings and ours may lie in the different types of brain activity measurable from MEG versus fMRI, or it could be due to the use of a different experimental task, as Schell et al. used a kind of grammaticality judgment task, which could lead to an increase in syntactic prediction, as syntactic prediction is a beneficial strategy in grammaticality judgment tasks and has been associated with greater activation in IFG due to increases in verbal working memory load (Matchin et al., 2017). It is also possible that, as meaning composition was always possible in this study, combinatorial processing was always active, masking any effects of the syntactic manipulation. In a direct comparison of lexico-semantic and syntactic composition, Fedorenko et al. (2020) found that no brain regions were selectively sensitive to syntax over semantic processes, and they suggest that combinatorial processes may not be fully distinguishable between syntactic and semantic/plausiblity manipulations.

We also found that although semantic plausibility alone was not a pre-condition to eliciting LATL conceptual combination effects, semantic plausibility led to an additional increase in LATL activation compared to the control in the same time window as conceptual combination. The factorial analyses, however, failed to identify semantic plausibility as a significant factor within this time window. The effects we did observe for semantic plausibility were about 100 ms later and replicated expected effects of this manipulation. Due to the timing of the identified temporal cluster associated with the main effect of plausibility, and due to the localization of the effect near the middle temporal gyrus, it is most likely that we are observing lexical access related to M350/N400 type activity (Maess et al., 2006; Pylkkänen & Marantz, 2003). This effect is well-studied and completely expected given our experimental design. The fact that we do not observe that plausibility independently modulates conceptual combination is consistent with the idea that a phrase must first be composed in order to assess if it is plausible given world knowledge (Pylkkänen et al., 2009) and consistent with recent work by Graessner et al. (2021), who observed a dissociation between phrasal composition and “meaningful composition” driven by a plausibility manipulation.

Neither syntactic composition nor semantic plausibility were observed to be *necessary* for conceptual combination, as one of the syntactic non-composition conditions and one of the semantically implausible conditions showed significantly greater activation than a non-combinatory control in the LATL and in a time window consistent with previous findings for conceptual combination. However, the implausible + syntactic non-composition condition did not show a statistically significant difference from the non-combinatory control. This finding should not be taken as evidence that this condition was itself non-combinatory, as a failure to reach significance alone is not an argument *in favor of* the null hypothesis. It may be that there was a greater degree of variance in this condition due to individual differences between participants that led to the lack of a significant difference from the control. However, we take the view that both syntactic constituency and semantic plausibility show additive effects in the activation patterns in the LATL alongside conceptual combination. Thus the lack of both syntactically combinable features and semantically compatible features may have rendered this condition indistinguishable (statistically) from the control, despite the fact that the implausible + syntactic non-composition condition appears visually to pattern with the plausible + syntactic non-composition condition. We further propose that this additive effect is responsible for the only condition that was fully well-formed at the target word, the plausible + syntactic composition condition, showing significantly greater activation than many of the other test conditions.

Considering that the plausible + syntactic composition condition was well-formed on the target word and that this effectively created a very brief garden path effect, the lack of any significant differences between conditions on the post-target word may be somewhat surprising. Many ERP studies have reported that reanalysis effects triggered by garden path sentences elicit a parietal positivity starting around 400–600 ms after the point of reanalysis (e.g., Gouvea et al., 2010; Kaan & Swaab, 2003), referred to as *the P600 effect*. However, many others have noted that “reanalysis” is a rather broad explanation for the P600 (Bornkessel et al., 2003; Tanner et al., 2017), and semantic anomalies can also elicit this component (Kim & Osterhout, 2005, et seq.). Under a similar account, in our study, the plausible + syntactic composition condition in the noun manipulation would have required syntactic reanalysis on the post-target word (*drinker*), and the implausible + syntactic composition condition may have required semantic reanalysis on the post-target word in order to arrive at the interpretation that the *drinker* is *impolite*, rather than the *tea*. However, we did not observe any significant effects of the syntactic or plausibility manipulations on the post-target word. This may be because the garden path effect in our study was much less pronounced than what has been used in previous studies. Many of the existing garden path effects relate to anomalies at the level of the verb, whereas in our study the temporary anomaly was restricted to the noun phrase. Given the argument structure and thematic role related steps necessary at the verb phrase, it may be that we did not create as strong a garden path effect.

Given the well-attested predictability effects that often co-occur with studies of semantic plausibility (and which also co-occur in this study), we can also conclude that lexical predictability did not appear to directly modulate conceptual combination in our study. Indeed, our direct manipulation of lexical probability did not have any effect on LATL conceptual combination and only appeared as a non-significant later effect on the post-target word. Note, however, that in this particular design, the contrast between high and low transitional probability was a relative one: “low” transition probability phrases had a corpus frequency of zero, while “high” just had to have a probability of greater than 0.05. Whether very highly predictable phrases still show effects of conceptual combination remains a topic for further study, as pre-activation of a lexical item in a highly predictable context could still interact with composition effects. Though we also observed a significant effect of word category on the post-target word characterized by greater activation for the noun manipulation (*tea drinker*) compared to the adjective manipulation (*sunny day*), as this was a factor in our analysis, we caution against any interpretation of those findings based on this study. First, the lexical differences between the noun and adjective manipulations were not very controlled in our stimuli: All the post-target words in the adjective condition were either plural nouns or mass nouns, while in the noun manipulation half were plural and half were singular. Additionally, we did not control for frequency differences on the post-target word between these two conditions. There were also multiple systematic differences between the noun and adjective manipulations, any of which could be responsible for the observed effect: (i) the noun manipulation had more morphologically complex words, as they were all deverbal nouns; (ii) the noun manipulation had more complex argument structure than the adjective manipulation, as the target word could always be interpreted as having an object relationship with the post-target word (a *tea drinker* is someone who *drinks tea*); and (iii) the adjective manipulation used adjectival modification, while the noun manipulation used nominal modification to form noun-noun compounds. Though the question of which differences in processing arise from different types of modification or of different levels of complexity in modification relationships is a highly relevant question, the current design does not allow us to shed any light on the topic, and we leave this question for future work.

### Conclusion

We observed that conceptual composition as reflected in the LATL shows a stronger activation pattern when there is local syntactic composition, though the lack of a syntactically licit parse does not completely eradicate the composition effect. This study supports a processing model where conceptual composition as reflected by LATL activity is influenced by local syntactic properties, but is able to operate independent of them. Further, we found no evidence that the local plausibility of the combination drives conceptual combination, supporting a processing account where conceptual composition is independent of semantic plausibility.

## ACKNOWLEDGMENTS

This work is funded by New York University Abu Dhabi Research Institute under Grant G1001. We thank Brian McElree and Alec Marantz for detailed feedback on earlier drafts, as well as the members of the Neuroscience of Language Lab at NYU and NYUAD for helpful discussion and feedback throughout. We also thank our two anonymous reviewers for their comments and suggestions.

## FUNDING INFORMATION

Liina Pylkkänen, New York University Abu Dhabi Research Institute Grant, Award ID: G1001 (https://dx.doi.org/10.13039/100012025).

## AUTHOR CONTRIBUTIONS

**Alicia Parrish**: Conceptualization; Investigation; Data curation; Formal analysis; Visualization; Writing – original draft; Writing – review & editing; Methodology; Project administration. **Liina Pylkkänen**: Conceptualization; Supervision; Writing – review & editing; Resources; Methodology; Funding acquisition.

## TECHNICAL TERMS

Syntactic composition: The process of combining syntactic categories to create a larger syntactic structure.
Conceptual combination: The process of combining two or more conceptual representations to create a more complex conceptual representation.
Plausibility: The degree to which the meaning of an expression describes a situation or circumstance that could happen in the real world.
